# Biomedicine and Biotechnology: Public Health Impact

**DOI:** 10.1155/2014/524785

**Published:** 2014-04-08

**Authors:** Nirmal K. Ganguly, Simon Croft, Lalji Singh, Subrata Sinha, Tanjore Balganesh

**Affiliations:** ^1^Translational Health Science and Technology Institute (THSTI), Gurgaon, India; ^2^The Institute of Liver and Biliary Sciences (ILBS), India; ^3^Immunology and Infection Department, Faculty of Infectious and Tropical Diseases, London School of Hygiene and Tropical Medicine, UK; ^4^Banaras Hindu University, Varanasi, Uttar Pradesh 221 005, India; ^5^National Brain Research Center, NH-8, Manesar, Gurgaon, Haryana 122 051, India; ^6^CSIR Centre for Mathematical Modelling and Computer Simulation (C-MMACS), NAL Belur Campus, Bangalore 560037, India

The goal of humankind is to alleviate the suffering from disease and quest is to have good and robust health always. The gain of knowledge through research is one of the primary steps towards the same. With the newly emerging infectious diseases, increase in the antimicrobial resistance and also the increasing incidences of noncommunicable diseases have thrown at us new challenges to develop better diagnostics, drugs, medical devices, and vaccines.

The present century is the era of biotechnology and information technology. Biotechnology has become the amalgamation of several technologies ranging from the field of biomedical research and synthetic biology and further transcending to engineering, nanotechnology, and bioinformatics. Biomedicine have gone a step further converging biology, chemistry, and physics which have led to increasing the understanding of the pathophysiological processes by deciphering the molecular interaction that plays a significant role in the cellular mechanism and thus devising new strategies to produce new diagnostics and therapies.

Health innovation process needs a boost to stem the declining productivity and high turnover rates of drugs with escalating cost. So, the promotion of newer technologies and innovation is the need of the hour. It is imperative to have health innovation reach the masses and add a value to the public health system of nations across the globe. With adequate impetus we are ushering in today the era of knowledge driven, evidence-based innovation in fields of biotechnology and biomedicine, leading to development of platform technologies which can have global implications.

This special issue in the journal of Biomed Research is a step towards the path which brings us to the forefront of new innovations and technologies that transcend all aspects of translational research and have high significance in the public health arena. Hence the editors have selected the cutting edge research in the field of medical biology which has bearing in the future of science.

The paper by Y. Qin et al., which have found that DNA vaccine encoding BCR/ABL-hil2 enhances the in vivo humoral and cellular responses in BABL/c mice, hence presenting a new targeted immunotherapy approach which holds promising finding for patients with chronic myeloid leukemia (CML). The paper postulates these findings and if the research is translated to effective DNA vaccine in humans for CML patients, it will help in the treatment of residual disease after the treatment with chemotherapy or targeted therapy.

New research in antimicrobial therapy has become a very essential tool to fight infection in context of growing antibiotic resistance. The paper by J. J. G.-R. Rodríguez et al., has given alternate strategy with cloned antimicrobial peptide PaDef homologous to defensins from Mexican avocado (*Persea americana* var.* drymifolia*). This paper reports the antimicrobial activity of defensin from avocado against* Escherichia coli* and* Staphylococcus aureus*. The alternative method of pathogen control paves the way for development of new treatment regimes against infectious pathogens.

The effect of curcumin from* Curcuma longa* L. (from the family Zingiberaceae) on fighting oxidative stress in liver and brain in mice fed with excessive alcohol was studied in the paper by C. W. Pyun et al. The finding showed that curcumin increases brain and hepatic phosphatidylcholine hydroperoxide levels in mice after consumption of excessive alcohol, hence proving the effectiveness of consumption of daily curcumin intake in protecting the liver and brain against alcohol induced oxidative stress.

The G. C. Fontes et al., paper on characterization of alginate and OSA starch bead for the use of controlled release carrier for penicillin is an important finding to alleviate the discomfort of patients undergoing conventional administration of the vital drug. This paper also has long implications on designing public health intervention for delivery of essential drugs.

The paper by M. P. L. Cunha et al., is the analysis of the vaccine adverse event reporting in the state of Rondonia, Brazil during the first ten years (1998–2008) after introduction of vaccines for BCG (Bacillus Calmette-Guerin), DTwP/Hib (hepatitis B, diphtheria, tetanus, pertussis + hemophillus influenza b), DTP (diphtheria, tetanus, and pertussis), MMR (measles, mumps, and rubella) and yellow fever (YF) is a major impact paper on public health. This study is of paramount importance which can help the regulators and clinicians alike from all over the world in gauzing the effectiveness and adverse reaction that can be anticipated from the regular vaccines that are a part of immunization programs of many countries around the world.

The simple model to analyze assessment of the antitoxin antibodies in the paper by A. Skvortsov and P. Gray also is an essential scientific finding which was once validated experimentally will be a very useful tool to assess in vitro the potential of protective antibodies for further evaluation in vivo.

The compilation of the articles in the special issue is very good read of high impact research and important scientific findings would have robust impact on strategizing innovative solutions of public health interventions globally; hence, all editors have selected the most promising research for publication.

*Nirmal K. Ganguly* *Simon Croft* *Lalji Singh* *Subrata Sinha* *Tanjore Balganesh*

